# Predicting Long-Term Respiratory Outcomes in Premature Infants: Is It Time to Move beyond Bronchopulmonary Dysplasia?

**DOI:** 10.3390/children7120283

**Published:** 2020-12-10

**Authors:** Deepak Jain, Alexander Feldman, Subhasri Sangam

**Affiliations:** Division of Neonatology, Department of Pediatrics, Rutgers Robert Wood Johnson Medical School, New Brunswick, NJ 08901, USA; feldmaam@rwjms.rutgers.edu (A.F.); subhasri.sangam@rutgers.edu (S.S.)

**Keywords:** preterm, bronchopulmonary dysplasia (BPD), respiratory morbidities, pulmonary function test

## Abstract

Premature birth has been shown to be associated with adverse respiratory health in children and adults; children diagnosed with bronchopulmonary dysplasia (BPD) in infancy are at particularly high risk. Since its first description by Northway et al. about half a century ago, the definition of BPD has gone through several iterations reflecting the changes in the patient population, advancements in knowledge of lung development and injury, and improvements in perinatal care practices. One of the key benchmarks for optimally defining BPD has been the ability to predict long-term respiratory and health outcomes. This definition is needed by multiple stakeholders for hosts of reasons including: providing parents with some expectations for the future, to guide clinicians for developing longer term follow-up practices, to assist policy makers to allocate resources, and to support researchers involved in developing preventive or therapeutic strategies and designing studies with meaningful outcome measures. Long-term respiratory outcomes in preterm infants with BPD have shown variable results reflecting not only limitations of the current definition of BPD, but also potentially the impact of other prenatal, postnatal and childhood factors on the respiratory health. In this manuscript, we present an overview of the long-term respiratory outcomes in infants with BPD and discuss the role of other modifiable or non-modifiable factors affecting respiratory health in preterm infants. We will also discuss the limitations of using BPD as a predictor of respiratory morbidities and some of the recent advances in delineating the causes and severity of respiratory insufficiency in infants diagnosed with BPD.

## 1. Introduction

Advances in the field of obstetrics and neonatology during the last half century have not only resulted in significant reduction in perinatal morbidities and mortality but also have led to an increase in survival of infants born at the limits of viability. The resultant increasing survival of more and more preterm infants into childhood and adulthood has brought new challenges for physicians and researchers alike, with increasing focus on reducing longer term morbidities. As more preterm infants are born during early stages of lung development, one of the major challenges has been delineating the impact of prematurity from the impact of lung injury on long-term respiratory morbidities. This information is crucial when evaluating the role of different perinatal preventive or treatment strategies in improving respiratory outcomes. Another key challenge has been to develop a more contemporary definition of bronchopulmonary dysplasia (BPD) to not only better reflect the degree of pulmonary dysfunction but also provide superior delineation of lung pathology, thereby potentially better predict longer term respiratory outcomes [1,2].

## 2. Long-Term Respiratory Outcomes in Premature Infants

Despite significant reduction in the severity of lung disease in premature infants born in the current era of high antenatal steroid (ANS) and surfactant use, and less invasive ventilation, these infants continue to have significant respiratory morbidities in infancy and childhood [3]. Premature infants have been shown to be prone to recurrent hospitalization secondary to lower respiratory tract infections, more frequently have respiratory symptoms such as wheezing, and are more likely to receive bronchodilators [4,5]. These morbidities are more frequent in infants of lower gestational age as well as those diagnosed with BPD [6,7,8,9]. Although the rate of hospitalizations gradually decreases with age, respiratory symptoms continue to be more common in adolescents born preterm with BPD when compared to full-term controls [8]. There is increasing evidence that even late preterm infants without significant lung injury in the perinatal period have increased risk of respiratory morbidities compared to their full-term counterparts [10,11].

One of the critical steps towards evaluating the effect of early life events on longer term lung health is to define the normal reference ranges for pulmonary function tests and their progression with age in healthy individuals. Recently, databases of pulmonary function test results from healthy individuals at different ages have been developed [12]. These data have enabled investigators to develop expected trajectories of pulmonary function tests through childhood to adults. In healthy individuals, forced vital capacity (FVC) and forced expiratory volume in 1 s (FEV_1_) increase through childhood to about 25 years of age, followed by gradual decline through to old age. The ratio of FEV_1_/FVC declines through life except for a small increase during adolescence [13].

As more preterm infants survive into young adulthood, there is increasing information available on the effect of prematurity as well as early lung damage on pulmonary function. The majority of these infants show an obstructive pattern of lung disease with significantly lower FEV_1_ and FEV_1_/FVC ratio in comparison to controls [8,14]. While these abnormalities are usually worse in adults with a history of BPD [15,16], even late preterm infants without significant lung disease also show deficits in lung functions in adolescence and adulthood [17,18]. There are contradicting results regarding the longitudinal progression of airflow limitation, with some studies suggesting improvement in pulmonary function over time whereas others suggest progressive worsening of airflow limitation potentially reflecting the degree of perinatal lung disease in the study population [19,20,21]. There is some evidence that severity of airflow limitation during early childhood could not only be predictive of lower pulmonary function in later life but also may predict increased risk of respiratory morbidities [22,23]. Over time, it is possible that pulmonary function in preterm infants follows similar trajectories as their full-term counterparts but with a lesser degree of peak in pulmonary function and potentially a more rapid decline in old age [21,24,25].

In terms of exercise capacity, the studies evaluating exercise capacity in adults born preterm have shown contradicting results with one study showing mild reduction in moderate to vigorous physical activity in children born at less than 32 weeks gestational age when compared to controls, whereas other studies have failed to show any significant difference [26,27,28]. Some of the limitations for the current evidence have been different measures of exercise capacity, relatively small sample size, and selection bias towards inclusion of a relatively healthier preterm population.

## 3. Factors Affecting Pulmonary Outcomes in Premature Infants

**Figure 1:**
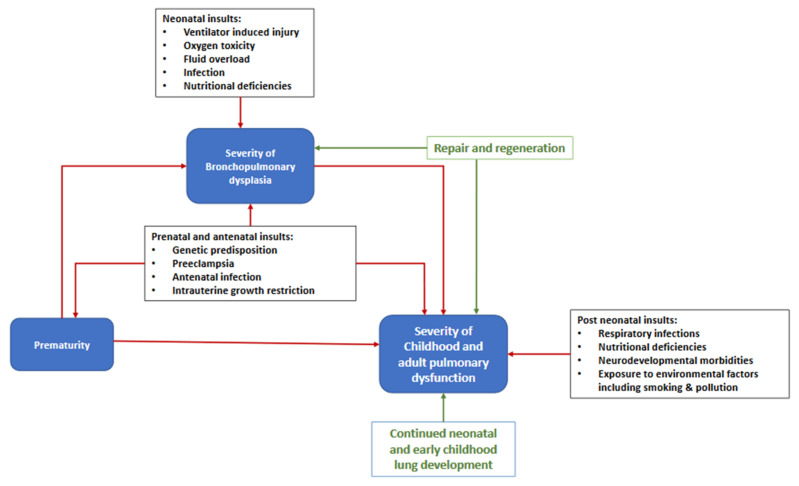
Algorithm for factors contributing to childhood and adult pulmonary dysfunction in premature infants.

The respiratory outcome in preterm infants represents an overall effect of multiple adverse factors including inherent genetic predisposition, various antenatal, neonatal and post-neonatal insults, balanced against regeneration, repair and continued alveolar development (Figure 1). While many of these factors are common to the pathogenesis of BPD, some of them may affect long-term respiratory outcomes independent of their effect on BPD.

### 3.1. Prematurity

Prematurity is an independent risk factor for adverse pulmonary outcomes. The fetus is exposed to relatively hypoxic conditions in-utero, which is crucial for normal airway, alveolar and vascular development [29]. Therefore, exposure of preterm lung to room air after preterm birth represent a relatively hyperoxic environment potentially resulting in lung injury and impaired lung development. As reported earlier, there is significant evidence that premature infants without the neonatal diagnosis of BPD or even late preterm infants have worse pulmonary outcome when compared to infants born at term gestation [11,30,31].

### 3.2. Prenatal and Antenatal Insults

In addition to genetic predisposition, early life events such as prenatal and antenatal insults play a key role in determining long-term health including the risk of pulmonary morbidities such as chronic obstructive lung diseases [32]. Some of these effects are not only limited to the exposed offspring but are transmitted to subsequent generations who have never been exposed to these insults [33]. These factors may act by altering the gene expression through epigenetic modifications including DNA methylation, histone modifications, chromatin structure and selected non-coding RNAs [34,35,36].

Maternal smoking prenatally and during pregnancy is one of the risk factors for poor lung function and respiratory morbidities in later life with several large cohort studies showing reduced pulmonary function in children and adults with in-utero smoke exposure [37,38,39]. Experimental studies in various animal models have shown that in-utero exposure to nicotine leads to abnormalities in airway branching, increased smooth muscle thickening and collagen deposition with some evidence of reduced alveolarization and capillary formation [40,41,42]. Some of the cellular pathways involved in these changes include alterations in Wnt and peroxisome proliferator-activated receptor gamma signaling at the alveolar and airway level, oxidative stress, alteration in hypothalamic–pituitary–adrenal axis, as well as epigenetic alterations including DNA methylation changes. In addition to attempts at smoking cessation, antioxidant treatment has been explored to mitigate the risk of tobacco exposure [41]. In a recent randomized controlled trial (RCT), McEvoy et al. showed that supplemental Vitamin C intake by pregnant smoker mothers resulted in significant reduction in wheezing and improved pulmonary function in infants at one year of age [43].

Intra uterine growth restriction (IUGR) is one of the important factors contributing to the fetal programming of later onset pulmonary diseases. Since IUGR is a fetal manifestation which could be due to multiple causes such as nutritional deficiency, placental insufficiency, chronic hypoxia or exposure to nicotine, the resultant abnormalities are likely to vary according to the insult. In preclinical studies in multiple animal models, IUGR has been shown to result in impaired alveolarization and vascularization, thickened septa, and reduced surfactant activity [44,45,46,47]. Multiple observational studies have documented reduced pulmonary function, namely, obstructive airway disease, and respiratory morbidities from infancy persisting into adulthood [48,49,50].

There are several other prenatal factors such as preeclampsia, maternal infection affecting the in-utero environment for lung development, in turn increasing the risk for long-term pulmonary morbidities. Whether these effects on long-term respiratory outcome are independent of preterm birth, lung damage and associated increase in the risk for BPD has not been firmly established.

### 3.3. Neonatal Insults

Premature infants are exposed to a multitude of factors, some of which are life sustaining but adversely affect normal lung development and hence increase the risk of respiratory morbidities. Over the last half century, advances in perinatal care has resulted in significant mitigation of these risk factors, but prematurity continues to be the strongest risk factor for poor respiratory health. Oxygen and positive pressure ventilation (which continue to be the most common BPD therapies) have been shown to adversely affect pulmonary outcome. Exposure of the developing lung to hyperoxia results in generation of reactive oxygen species, thereby resulting in cell injury and apoptosis, increased inflammation and pulmonary edema. Multiple preclinical studies in different animal models have documented disruption of alveolar development, fibrosis, and increased airway resistance after exposure to hyperoxia. Mechanical ventilation results in lung injury by overstretching of tissues by overinflation (Volutrauma) and repeated opening of close lung units (atelectotrauma). Both have been shown to result in cell injury, activation of inflammatory cascade, and pulmonary edema, in turn increasing the need for positive pressure ventilation resulting in a vicious cycle. There are several other risk factors such as postnatal infection, increased pulmonary blood flow because of left to right shunt through patent ductus arteriosus, or nutritional deficiencies in the neonatal period that play an important role in lung damage, thereby impacting long-term pulmonary outcomes.

### 3.4. Post-Neonatal Factors

After discharge from hospital, long-term pulmonary health in preterm infants is the end result of the balance between factors encouraging alveolar growth and repair, competing against continued exposure to injurious stimuli through childhood and adults. Childhood respiratory infection, such as respiratory syncytial virus, may result in further lung damage and continues to be one of the risk factors for increased pulmonary morbidities and abnormal lung function [51,52]. Multiple studies have shown the impact of postnatal nutrition on lung function with breast feeding and adequate vitamin supplementation having a positive impact on lung volume and pulmonary function [53,54].

In addition to antenatal period, there is some evidence that exposure to tobacco smoke in childhood and beyond is an independent risk factor for adverse pulmonary outcome, independent of antenatal exposure [55]. One of the limitations of the current evidence is the lack of adequately powered studies with subjects only exposed to tobacco smoke postnatally [56]. In addition to tobacco smoke, early childhood and adult exposure to environmental pollutants such as oxidant gasses, traffic related emissions, and particulate matter have been shown to result in increased risk of respiratory morbidities and reduced lung function [57,58,59,60]. A significant reduction in exposure to environmental toxins can potentially improve long-term respiratory outcomes.

### 3.5. Post-Neonatal Lung Development and Lung Function Catch-Up

There is an increase in alveolar number after birth which has been previously thought to be completed by 2 years of age. However, there is some evidence that alveolar growth may continue beyond infancy through adolescence [61]. The evidence for functional improvement has been suggested by some of the longitudinal studies demonstrating some degree of catch up in pulmonary function with age, which in some patients can reach up to the lower range of normal [62,63]. The degree of this catch-up growth is modified by environmental factors [64]. There is some early evidence of using pharmacological interventions such as stem cells to promote alveolar development or drive lung repair [65,66].

## 4. Bronchopulmonary Dysplasia and Pulmonary Outcomes

**Figure 2:**
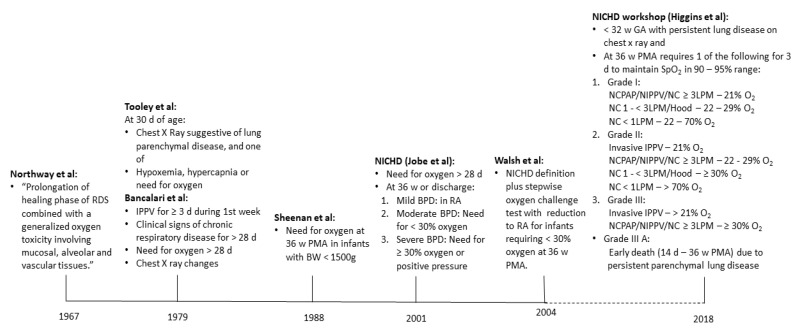
Timeline of evolution of bronchopulmonary dysplasia (BPD) definitions. NICHD: Eunice Kennedy Shriver National Institute of Child Health and Human Development; PMA: post menstrual age; RDS: Respiratory Distress Syndrome; IPPV: intermittent positive pressure ventilation; BW: Birth Weight; RA: room air; NCPAP: nasal continuous positive airway pressure; NIPPV: noninvasive positive pressure ventilation; NC: nasal cannula; LPM: liter per minute.

BPD is a clinical diagnosis used to define preterm infants with chronic lung disease who are likely to be at high risk of adverse pulmonary outcomes. It is one of the rare conditions where a disease is defined by the need for the treatment acting as a surrogate for the degree of pulmonary dysfunction. As described by Northway and colleagues in 1967, the term BPD was used to describe the chronic phase of lung disease following respiratory distress syndrome in preterm infants with significant radiographic changes [67]. Over time, as more preterm infants survived, the definition of BPD evolved into more of a clinical criterion of oxygen need at 28 days and then at 36 weeks [68,69]. In 2001, Jobe and Bancalari proposed the first comprehensive gradation of BPD severity based on oxygen and positive pressure support requirements at 36 weeks [70]. As clinical care practices evolved since then, these criteria were recently updated during the Eunice Kennedy Shriver National Institute of Child Health and Human Development (NICHD) workshop in 2016 [1]. Figure 2 summarizes the timeline of major changes in the definition of BPD.

### 4.1. Adolescent and Early Adulthood Pulmonary Outcomes in Infants with BPD

One of the key reasons for the changes in the definition has been the quest to better predict long-term pulmonary outcomes. As more preterm infants with BPD are surviving into adulthood, there is increasing information about longer term pulmonary outcomes in infants with BPD. In a recent publication, Doyle and colleagues described pulmonary function test results from 8 years to 25 years of age in a cohort of extremely preterm infants born between 1919 and 1992. In this cohort, at 25 years of age, infants with BPD had significant reduction in airflow compared to preterm infants without BPD (FEV_1_ % predicted 83.6 (14.3) in BPD vs. 91.6 (14.0); mean difference −8.0 (−12.0 to −4.0), *p* value <0.001. There was significant reduction in FEF _25–75%_ with no significant difference in FVC between the groups, with significant reduction in FEV_1_/FVC in BPD group. In addition, in subjects ranging from 8 years to 25 years of age, pulmonary function followed the similar trajectories in infants with BPD, infants without BPD, and controls, suggesting persistent effect on airflow obstruction into young adulthood [71]. Two other large cohorts, the EPICure study cohort of 11- and 19-year-old children born extremely preterm in the British Isles in 1995 and a cohort of 26- to 30-year-old adults born at a very low birth weight in New Zealand in 1986, who underwent pulmonary function tests and have shown similar results with worse airflow obstruction in infants with BPD when compared to infants without BPD [15,72]. In addition to the decrease in pulmonary function tests, these patients have been consistently shown to have increased incidence of asthma-like symptoms and reduced exercise tolerance potentially increasing the risk for chronic obstructive pulmonary disease [73,74,75].

### 4.2. Pulmonary Outcomes in Infants with New BPD

One of the limitations of the evidence for long-term pulmonary outcome in infants with BPD has been applicability of these results to the current population of extremely preterm infants with BPD, as these cohorts were born more than 15 to 20 years ago with limited use of antenatal corticosteroids (ACS) or surfactant and different respiratory support practices. This was reflected in an individual patient meta-analysis of the studies which showed less than half of subjects receiving ACS, surfactant administration in one fifth, and varying definitions of BPD used in the individual studies [76].

As expected, there are limited data on long-term outcomes of extremely preterm infants born at late canalicular and early saccular stages of lung development in the current era of high ACS and exogenous surfactant use, less invasive respiratory support practices. These infants are more likely to develop a newer form of BPD characterized by alveolar simplification with abnormal vasculogenesis but less airway damage, vascular remodeling and pulmonary hypertension [77,78]. Several researchers have evaluated pulmonary functions in school age children infants with new BPD and have demonstrated reduced diffusion capacity in addition to obstructive flow pattern in these infants when compared to preterm infants without BPD [79,80]. On the other hand, other studies evaluating pulmonary function in extremely preterm infants have demonstrated reduced lung volume and airflow with prematurity with limited added impact of BPD [81,82]. These infants and children continue to be at increased risk for hospitalizations, ICU admission, higher incidence of asthma or wheezing, and are treated with more bronchodilators [83,84,85]. These morbidities gradually decrease with age but the impact on longer term morbidities into adulthood is currently not known.

### 4.3. Outcomes in Infants with Very Severe BPD

With increasing survival of preterm infants at limits of viability, there has been increasing need to differentiate infants with a more severe form of lung injury requiring prolonged mechanical ventilation [86]. These infants are at high risk of death with surviving infants continuing to require significant respiratory support beyond 36 weeks post menstrual age (PMA). The clinical course of these infants is commonly complicated by severe pulmonary hypertension requiring pulmonary vasodilators, airway obstruction from tracheobronchomalacia, as well as poor nutrition and growth. After discharge, these infants are at particularly high risk for intensive care admission and mortality during early childhood and severe neurodevelopmental impairments [87,88,89]. Some of the risk factors associated with the adverse outcomes include the need for tracheostomy as well as other comorbidities such as pulmonary hypertension or control of breathing issues [90,91].

The data on the impact of very severe BPD on longer-term respiratory outcomes are relatively sparse due to low incidence and high mortality. In addition, the ability to reliably perform pulmonary function tests in this population continues to be a significant challenge. As increasing number of these infants survive into childhood, there is an urgent need for longer-term follow up in order to develop preventive and management strategies for this subset of the population with severe BPD.

## 5. BPD Definition as Predictor of Pulmonary Outcomes

As more preterm infants are surviving into adulthood, there is increasing impetus from researchers, parents, and policy makers to use longer term respiratory morbidities as a relevant outcome of interest for any preventive or treatment strategies [92]. Since measuring these outcomes can be cost prohibitive, time consuming and difficult to perform at a large scale, BPD has been commonly used as a surrogate for them. Though there is some evidence of correlation between the current severity-based definition of BPD and respiratory outcomes, the strength of this correlation has varied across the studies from strong to insignificant [16,93,94,95,96]. This is likely reflected in some of the recent evidence where well-studied perinatal practices known to decrease the incidence of BPD may not affect long-term respiratory outcome, whereas others with no effect on BPD may improve some longer-term pulmonary outcomes [3,97]. This observation has highlighted the limitations of the current definitions of BPD with renewed efforts to find a pathophysiology-based definition of BPD which may better reflect current patient population and neonatal practices.

There have been efforts to optimize the current definition of BPD by refining the diagnostic criteria. One of the challenges for defining BPD has been finding the right time of assessment. This was reflected in a retrospective study of about 1500 extremely preterm infants admitted to Canadian Neonatal Network neonatal intensive care units in which predictive accuracy of BPD for respiratory outcomes progressively improved as timing of assessment increased from 34 weeks to 40 weeks PMA [98]. In another study from the infants admitted to the NICHD neonatal network, Jensen and colleagues evaluated 18 different combinations of respiratory support and oxygen concentration at 36 weeks’ PMA to identify the definition to best predict death or respiratory and neurologic outcomes. Interestingly, the definition involving modes of respiratory support had the best predictive accuracy with no effect of inspired oxygen concentration [99]. The latest attempt to revise the definition of BPD has come from the workshop held by Eunice Kennedy Shriver NICHD in 2016, which suggested several changes including radiographic confirmation of parenchymal lung disease, including early death owing to persistent parenchymal lung disease and respiratory failure, target oxygen saturation ranges, as well as nasal cannula oxygen at different flow rates [1]. Though physiologically sound, there are currently no data about if these changes in definition improve predictive accuracy of longer-term outcomes.

Since the need for oxygen and respiratory support is likely to be imprecise in defining lung pathology, recently there has been efforts to better define lung disease in the infants with BPD. Some of these recent studies have utilized magnetic resonance imaging and chest computed tomography (CT) to develop a specific phenotype of lung disease and scoring systems for severity of lung disease [100,101]. In a small study in 42 neonates by Higano and colleagues, the magnetic resonance imaging (MRI) score correlated well with clinical severity and short-term respiratory outcomes [102]. Similarly, in a recent study in infants with severe BPD, Wu and colleagues used a combination of chest CT with angiography and echocardiography to differentiate the degree of parenchymal, airway and vascular disease [103]. These tools may not be ready for primetime use but could be potentially used in a group of the high-risk population to refine management strategies, as well as possibly reflect future use of precision medicine in neonatology [104]. In addition to structural differentiation, several efforts have been made to differentiate parenchymal lung disease by its effect on gas exchange. Shepherd and colleagues were able to classify a group of infants with severe BPD into obstructive, restrictive and mixed phenotypes by performing pulmonary function tests (PFTs) at about 52 weeks’ postmenstrual age [105]. Some of the other markers to define alteration in pulmonary function in infants with BPD include volumetric capnography [106], shift of oxyhemoglobin dissociation curve [107], or variability in tidal breathing parameters [108]. All of these markers need to be further evaluated for correlation with longer term outcomes in large cohorts before they can be recommended outside the research settings.

## 6. Conclusions

There is increasing information on early life origins of adult onset pulmonary diseases such as COPD with multiple prenatal, neonatal and childhood factors playing a critical role in the ability to achieve normal pulmonary function. Premature infants, especially those with significant perinatal lung injury resulting in BPD, are likely to achieve suboptimal pulmonary function during adult life, putting them at high risk of developing symptomatic lung disease. There is an urgent need to develop a pathophysiology and pulmonary function-based definition of BPD to help better predict longer term morbidities. As BPD is one of the multiple factors affecting long-term respiratory outcomes, albeit an important one, premature infants irrespective of the diagnosis of BPD should be closely followed for long-term respiratory health.
